# *Entransia* and *Hormidiella*, sister lineages of *Klebsormidium* (Streptophyta), respond differently to light, temperature, and desiccation stress

**DOI:** 10.1007/s00709-015-0889-z

**Published:** 2015-10-06

**Authors:** Klaus Herburger, Ulf Karsten, Andreas Holzinger

**Affiliations:** 1 Institute of Botany, Functional Plant Biology, University of Innsbruck, Sternwartestraße 15, A-6020 Innsbruck, Austria; 2 Institute of Biological Sciences, Applied Ecology and Phycology, University of Rostock, Albert-Einstein-Straße 3, D-18059 Rostock, Germany

**Keywords:** Green algae, Desiccation stress, Photosynthesis, Respiration, Temperature, Transmission electron microscopy

## Abstract

The green-algal class Klebsormidiophyceae (Streptophyta), which occurs worldwide, comprises the genera *Klebsormidium*, *Interfilum*, *Entransia*, and *Hormidiella*. Ecophysiological research has so far focused on the first two genera because they are abundant in biological soil crust communities. The present study investigated the photosynthetic performances of *Hormidiella attenuata* and two strains of *Entransia fimbriata* under light, temperature, and desiccation stress. Their ultrastructure was compared using transmission electron microscopy. The two *Entransia* strains showed similar physiological responses. They used light more efficiently than *Hormidiella*, as indicated by higher oxygen production and relative electron transport rate under low light conditions, lower light saturation and compensation points, and higher maximum oxygen production during light saturation. Their requirement for low light levels explains the restriction of *Entransia* to dim limnetic habitats. In contrast, *Hormidiella*, which prefers drier soil habitats, responded to light gradients similarly to other aero-terrestrial green algae. Compared to *Entransia*, *Hormidiella* was less affected by short-term desiccation, and rehydration allowed full recovery of the photosynthetic performance. Nevertheless, both strains of *Entransia* coped with low water availability better than other freshwater algae. Photosynthetic oxygen production in relation to respiratory consumption was higher in low temperatures (*Entransia*: 5 °C, *Hormidiella*: 10 °C) and the ratio decreased with increasing temperatures. *Hormidiella* exhibited conspicuous triangular spaces in the cell wall corners, which were filled either with undulating cell wall material or with various inclusions. These structures are commonly seen in various members of Klebsormidiophyceae. The data revealed significant differences between *Hormidiella* and *Entransia*, but appropriate adaptations to their respective habitats.

**Electronic supplementary material:**

The online version of this article (doi:10.1007/s00709-015-0889-z) contains supplementary material, which is available to authorized users.

## Introduction

The green-algal class Klebsormidiophyceae (Streptophyta) comprises the four genera *Klebsormidium*, *Interfilum*, *Entransia*, and *Hormidiella* (Leliaert et al. 2012) and occurs worldwide in freshwater and aero-terrestrial habitats (Rindi et al. 2011). Particularly, *Klebsormidium* and *Interfilum* have been studied intensively in recent years, because species of both genera are important components of biological soil crust communities (Mikhailyuk et al. 2008, 2015; Karsten and Holzinger 2014). In these microecosystems, which occur mainly in dry lands or disturbed environments, they contribute significantly to primary production (Karsten and Holzinger 2014), carbon and nitrogen cycling (Elbert et al. 2012), soil stabilization, and water retention (Evans and Johansen 1999). Therefore, much effort has been expended to investigate the strategies used by these streptophyte green algae to cope with the harsh environmental conditions of their terrestrial habitats (Gray et al. 2007; Karsten et al. 2010, 2013, 2014, 2015; Kouřil et al. 2001; Kaplan et al. 2012; Karsten and Holzinger 2012; Kitzing et al. 2014), with a focus on *Klebsormidium* (Holzinger and Karsten 2013 and references therein), the largest genus of the Klebsormidiophyceae (Ryšánek et al. 2015). Transcriptome and genome analyses of *Klebsormidium crenulatum* and *Klebsormidium flaccidum* (Holzinger et al. 2014; Hori et al. 2014) have shown that these species contain several genes that are specific to land plants, involved in hormone signaling (Holzinger and Becker 2015) and cellular responses to desiccation stress. Thus, the members of Klebsormidiophyceae are also interesting in an evolutionary context, as land plants evolved from streptophyte green algae (Lewis and McCourt 2004).

In a recent study, four strains of *Interfilum*, from biogeographically different soil habitats representing a gradient of annual precipitation (388 to 1162 mm) were investigated in regard to their desiccation tolerance as well as their light and temperature requirements for photosynthesis (Karsten et al. 2014). The authors showed that these four strains exhibited certain fundamental traits that seem to be important for an aero-terrestrial lifestyle, such as high photophysiological plasticity under variable photon fluence rates. This plasticity can be found in the closely related *Klebsormidium*, and also in other aero-terrestrial streptophyte green algae (Hawes 1990; Elster and Benson 2004; Aigner et al. 2013; Kaplan et al. 2013; Vilumbrales et al. 2013; Pichrtová et al. 2014a, b; Herburger et al. 2015). On the other hand, various strains of *Interfilum* showed significantly different kinetics in photosynthetic signal loss in response to experimentally applied desiccation stress, as well as under gradients of increasing temperature and light, compared to each other and to several strains of *Klebsormidium* (Karsten et al. 2010, 2013; Karsten and Holzinger 2012). This raises the question of whether strains belonging to other genera of the Klebsormidiophyceae (*Entransia*, *Hormidiella*) show similar ecophysiological response patterns to the stressful environmental conditions prevailing in terrestrial or limnetic transitional habitats. However, such ecophysiological data on *Entransia* and *Hormidiella* are very limited. The effect of exposure to ultraviolet radiation (UVR) on members of these two genera and on *Klebsormidium*/*Interfilum* was recently investigated (Kitzing et al. 2014; Kitzing and Karsten 2015), showing that all tested *Klebsormidium* and *Interfilum* strains synthesize and accumulate the same UV-sunscreen compound as a photoprotectant, when exposed to enhanced UVR. While *Hormidiella* is also able to synthesize and accumulate a different specific UV-sunscreen compound from that in *Klebsormidium* and *Interfilum*, *Entransia* does not contain and is also not capable of accumulating such a solute under UVR (Kitzing and Karsten 2015). The lack of a UV-sunscreen in *Entransia* was accompanied by a strong reduction in photosynthetic activity when exposed to UVR (Kitzing and Karsten 2015), indicating fundamental differences in the biochemical properties and ecophysiological response patterns in the four related genera of Klebsormidiophyceae. Beyond these few physiological data, knowledge of *Entransia* and *Hormidiella* is restricted to information from their original descriptions (Iyengar and Kanthamma 1940; Hughes 1948; Subrahmanyan 1976), phylogenetic analyses (McCourt et al. 2000; Sluiman et al. 2008), and morphological or ultrastructural studies of filaments during asexual reproduction (Lokhorst et al. 2000; Cook 2004).

In this study, the photosynthetic performance of two strains of *Entransia fimbriata* (UTEX2353, UTEX2793) and one strain of *Hormidiella attenuata* (CCAP329/1) was evaluated under conditions of increasing light and temperature gradients and desiccation stress. The filamentous algae were obtained from different limnetic transitional (UTEX2353, UTEX2793) and soil (CCAP329/1) habitats from the USA, Canada, and Brazil. We hypothesized that (1) *Entransia* shows different response patterns to light, temperature, and desiccation stress compared to aero-terrestrial members of the Klebsormidiophyceae, as this genus is restricted to rather humid environments; and that (2) the response of *Hormidiella* is more comparable to those of algae from other aero-terrestrial habitats (*Klebsormidium*, *Interfilum*). We further investigated whether the physiological performance of *Entransia* and *Hormidiella* exhibits traits that are common in Klebsormidiophyceae or can be explained by their different habitats. Finally, the morphology and ultrastructure of the three strains were compared using light and transmission electron microscopy (TEM).

## Material and methods

### Strain origin and culture conditions

UTEX2353 and UTEX2793 (*E. fimbriata*) were obtained from ‘The Culture Collection of Algae at the University of Texas at Austin (UTEX).’ CCAP329/1 (*H. attenuata*) was purchased from ‘The Culture Collection of Algae and Protozoa (CCAP)’ and an additional sample was kindly provided by A. Lukešová (Institute of Soil Biology, AS CR, Czech Republic). Details of the original habitat, including meteorological data, and species assignment are listed in Table 1. Algae were cultured in 250-mL Erlenmeyer flasks in modified BBM (3 NMBBM; Starr and Zeikus 1993) under the same conditions as described by Karsten et al. (2014). For microscopic examination and physiological measurements, cell filaments from 4-week-old cultures were used.

**Table 1:** Characterization of two *Entransia fimbriata* strains (UTEX2353, UTEX2793) and *Hormidiella attenuata* (CCAP329/1)

Strain number	Habitat	Meteorological data	Species assignment and authority; sequence accession (rbcL, SSU DNA, group I intron, ITS1, ITS2 rRNA)
UTEX2353	Littoral zone of Fawn Lake (~340 m a.s.l.), Ontario, Canada; isolated 1981 by N. Nakatsu	Min. air temperature: −20–15 °C Max. air temperature: −10–25 °C Monthly rainfall days: 12–19 Monthly precipitation: 18.5–123.1 mm Annual rainfall: 756.5 mm	McCourt et al. 2000; Karol et al. 2001, Turmel et al. 2002, Sluiman et al. 2008, Rindi et al. 2011; AJ549226
UTEX2793	*Sphagnum* bog on Bird Lake Rd (~490 m a.s.l.), Oneida Co., Wisconsin, USA; isolated 2001 by C.F. Delwiche	Min. air temperature: −16–12 °C Max. air temperature: −5–25 °C Monthly rainfall days: 8–16 Monthly precipitation: 17.7–123 mm Annual rainfall: 879 mm	Sluiman et al. 2008; Cook 2004; AY823714
CCAP329/1	Soil from a xeromorphic forest (cerradão; ~800 m a.s.l.), São Carlos, São Paulo State, Brazil; isolated 1996 by J. Komárek	Min. air temperature: 13–20 °C Max. air temperature: 25–30 °C Monthly rainfall days: 3–11 Monthly precipitation: 30.8–267.2 mm Annual rainfall: 1495 mm	Lokhorst et al. 2000; Sluiman et al. 2008, Rindi et al. 2011; AM419033

Strain number, habitat characteristics, meteorological data (temperatures expressed as means; www.worldweatheronline.com, www.climatemps.com), and species assignment including GenBank accession numbers are given

### Light microscopy

Algal filaments were investigated with a Zeiss Axiovert 200 M microscope, equipped with a 63 × 1.4 NA objective and an Axiocam MRc5 camera controlled by Zeiss Axiovision software. Contrast was enhanced by using differential interference contrast (DIC). All images were further processed with the software Adobe Photoshop (CS5) version 12.1 (Adobe Systems, San José, CA, USA).

### Transmission electron microscopy

TEM was performed according to Holzinger et al. (2009) with modifications. Briefly, filaments of UTEX2353, UTEX2793, and CCAP329/1 were fixed in 20 mM cacodylate buffer (1 h, pH 6.8) containing 2.5 % glutaraldehyde, and postfixed in 1 % osmium tetroxide (~18 h at 4.6 °C). After rinsing, probes were dehydrated in increasing ethanol concentrations and propylenoxid and embedded in modified Spurr’s resin. Ultrathin sections were prepared with a Reichert Ultracut (Leica Microsystems, Wetzlar, Germany) and counterstained with 2 % uranyl acetate and Reynold’s lead citrate. Sections were examined with a Zeiss Libra 120 transmission electron microscope (80 kV) connected to a ProScan 2 k SSCCD camera, controlled with OSIS iTEM software.

### Light dependence of photosynthesis and respiration (PI-curves)

Photosynthetic oxygen production and respiratory consumption in response to increasing photosynthetically active radiation (PAR) and darkness, respectively, were recorded according to Remias et al. (2010). A Presens Fibox 3 oxygen optode (Presens, Regensburg, Germany) was attached to a 3-mL thermostatic acrylic chamber (type DW1, placed on a magnetic stirrer to ensure homogeneous light absorption; Hansatech Instruments, Norfolk, UK). A connected Thermo Haake K20 refrigerated circulator (Thermo Fisher Scientific Inc., Waltham, MA, USA) ensured constant temperature (20 °C) during measurements. The chamber was filled with 3 mL algal suspension (UTEX2353, UTEX2793 or CCAP329/1) enriched with 2 mM NaHCO_3_ (final concentration) and exposed to ten increasing photon fluence rates (0–1000 μmol photons m^−2^ s^−1^) for 6 min each. Respiration (*R*) in the dark was measured for 6 min directly before and after the light measurements, and the mean was taken to express *R*. Subsequently, after each measurement, algae were immobilized on a Whatman GF/F glass microfiber filter (Ø 47 mm; Whatman, Dassel, Germany) to extract and quantify chlorophyll (chl.) *a* with 3 mL dimethyl formamide (DMF; Sigma-Aldrich, Steinheim, Germany) according to Porra et al. (1989). Oxygen production and consumption were expressed in μmol O_2_ h^−1^ mg^−1^ chl. *a*, and the calculated PI curves were fitted by the model of Webb et al. (1974) to derive four photosynthetic parameters: α, positive slope at limiting photon fluence rates (μmol O_2_ h^−1^ mg^−1^ chl. *a* (μmol photons^−1^ m^−2^ s^−1^)^−1^); *I*_c_, light compensation point (μmol photons m^−2^ s^−1^); *I*_k_, initial value of light-saturated photosynthesis (μmol photons m^−2^ s^−1^); and *P*_max_, maximum photosynthetic oxygen production in the light saturation range (μmol O_2_ h^−1^ mg^−1^ chl. *a*). To estimate whether high photon fluence rates cause photoinhibition, a pulse-amplitude modulated fluorimeter (PAM 2500, Heinz Walz GmbH, Effeltrich, Germany) was used to determine the relative electron transport rates (rETRs) as a function of increasing PAR up to 2015 μmol photons m^−2^ s^−1^ (Herburger et al. 2015). Algal filaments (UTEX2353, UTEX2793, or CCAP329/1) were placed in a KS-2500 suspension cuvette (Heinz Walz GmbH) and exposed to 17 increasing light steps (0–2015 μmol photons m^−2^ s^−1^, each 30 s). To avoid shading effects inside the cuvette, only a few algal filaments were used (~1–2 mg chl. *a* L^−1^). Actinic light was provided by a LED (630 nm), and the effective quantum yield of PSII [Y(II)] was determined by a saturation pulse analysis (Schreiber and Bilger 1993) after each light step. The calculated rETR curve (Kromkamp and Forster 2003) was fitted according to Walsby (1997), as slight photoinhibition occurred. This allowed us to derive the three photosynthetic parameters α, *I*_k_ (μmol photons m^−2^ s^−1^), and rETR_max_ (maximum electron transport rate). Linear regressions between the mean values of O_2_ production (μmol O_2_ h^−1^ mg^−1^ chl. *a*) and rETR at PAR 30, 55, 105 and 105, 200, 490, 1000 μmol photons m^−2^ s^−1^ were calculated to compare the kinetics of O_2_ and rETR curves.

### Temperature dependence of photosynthesis and respiration

The effect of changing temperatures on photosynthetic oxygen production and respiratory consumption was examined according to Karsten and Holzinger (2012). A thermostatic acrylic chamber connected to a Thermo Haake K20 refrigerated circulator was filled with 3 mL algal suspension (UTEX2353, UTEX2793 or CCAP329/1) enriched with 2 mM NaHCO_3_ and exposed to nine increasing temperatures (5 to 45 °C in 5 °C steps) at 200 μmol photons m^−2^ s^−1^. The O_2_ production (gross photosynthesis) and consumption (respiration) were referenced to the total amount of chlorophyll *a* per sample as described in the previous section (extraction by DMF). Additionally, net photosynthesis and gross photosynthesis:respiration (P/R) ratios were calculated for each temperature.

### Monitoring the effective quantum yield of PSII during desiccation and rehydration

The effect of desiccation and rehydration on the effective quantum yield of PSII [Y(II)] of UTEX2353, UTEX2793, and CCAP329/1 was estimated by using a specially designed desiccation chamber (Karsten et al. 2014). Algal filaments (~1–2 mg Chl *a* L^−1^) and 200 μL modified BBM were transferred to Whatman GF/F glass fiber filters (*n* = 4) and placed in the desiccation chamber filled with 100 g silica gel, which resulted in a relative air humidity (RH) of ~10 % inside the chamber. The RH was recorded with a PCEMSR145STH mini data logger (PCE Instruments, Meschede, Germany; Supplementary Fig. S1), and the chamber was exposed to constant light (40 μmol photons m^−2^ s^−1^) at ambient room temperature (22 ± 0.5 °C). The Y(II) determined by saturation pulse analysis of the desiccating algae was measured through the transparent top lid of the chamber by using a PAM2500, while the distance between the PAM light probe and the algal filaments was constant (12 mm). Once the Y(II) of each replicate of the individual algal strain reached 0, the desiccation period was stopped. This was immediately followed by rehydrating the algae on the filters with 200 μL of modified BBM and placing them in a chamber containing 100 mL tap water (RH ~96 %) to measure the recovery of the Y(II) for ~2 days.

### Statistics

Comparisons of photosynthetic parameters derived from light response curves (O_2_ (*n* = 3): α, *I*_c_, *I*_k_, *R*, *P*_max_; rETR (*n* = 4): α, *I*_k_, rETR_max_), effects of desiccation [Y(II), *n* = 4], or temperature (O_2_, *n* = 3) on photosynthesis as well as P:R ratios (*n* = 3) were performed by one-way ANOVA followed by Tukey’s post hoc test (*P* < 0.05) to find homogeneous subgroups of significantly different means. Comparison of the means of O_2_ production with rETR at PAR 30, 55, 105 and 105, 200, 490, 1000 μmol photons m^−2^ s^−1^ was performed by linear regression, and *R*^2^ was calculated (not shown). Data are presented as means and standard deviation. Analyses were carried out in Origin 8.5 (OriginLab Corporation, Northampton, MA, USA).

## Results

### Light microscopy

**Fig. 1:**
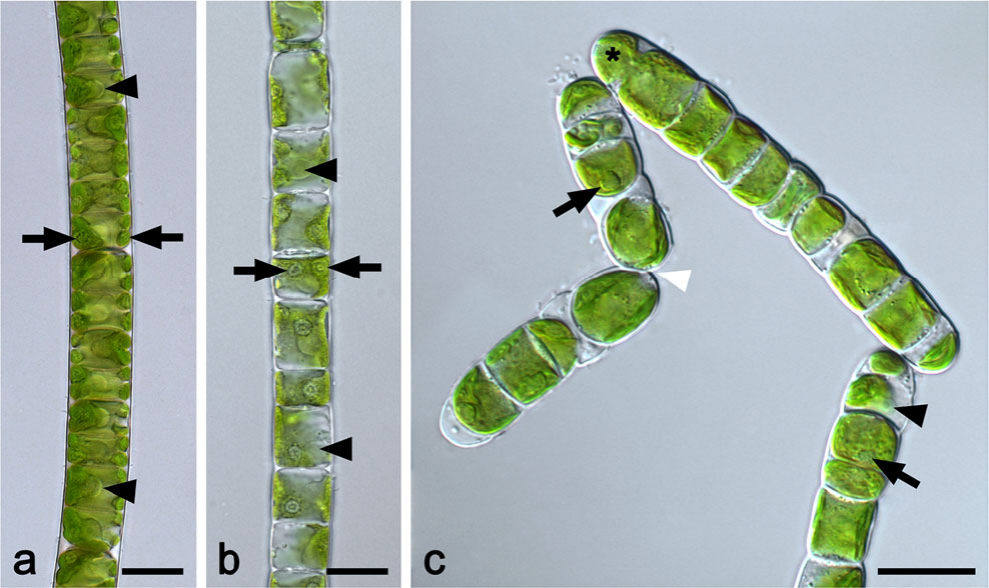
Cell filaments of **a** UTEX2353 (*Entransia fimbriata*), **b** UTEX2793 (*E. fimbriata*), and **c** CCAP329/1 (*Hormidiella attenuata*). **a** Cells contain one centrally located nucleus (*arrowheads*) and one parietal chloroplast covering the entire length of the cell and containing at least two pyrenoids (*arrows*). **b** The lobed parietal chloroplast is less expanded compared to **a**. Two or more prominent pyrenoids per chloroplast surrounded by starch grains (*arrows*). Cells contain one nucleus (*arrowheads*). **c** Detaching filaments (*white arrowhead*) with one parietal chloroplast per cell, containing one pyrenoid (*arrows*). Terminal cell narrowed toward polar end (*asterisk*); nucleus (*black arrowhead*). *Bars* 10 μm

Cells of strictly uniseriate filaments of both *Entransia* UTEX2353 and UTEX2793 contained one parietal chloroplast with lobed (sometimes fimbriate) longitudinal margins (Fig. 1a, b). Each chloroplast contained two or more pyrenoids surrounded by starch grains (Fig. 1a, b). In UTEX2353, the chloroplast covered the entire length of the cell (Fig. 1a), while it was less expanded in UTEX2793 (Fig. 1b). In both strains, the clearly visible nucleus was located in the center of the cell (Fig. 1a, b). Fragmentation into short filaments was observed only occasionally. Cell filaments of *Hormidiella* CCAP329/1 were uniseriate with one parietal chloroplast, which covered about two-thirds of the cell circumference, had smooth longitudinal margins, and extended to the cell cross-wall (Fig. 1c). A single pyrenoid surrounded by starch grains was embedded in each chloroplast (Fig. 1c). Fragmentation into short filaments (4–12 cells) was found frequently (Fig. 1c). The terminal cells of filaments narrowed toward the polar end (Fig. 1c). As in both *Entransia* strains, no hyaline stalk formed by a terminal cell and no reproductive stages (aplanospores and zoospores; Lokhorst et al. 2000; Cook 2004) were found in the culture.

### Transmission electron microscopy

**Fig. 2:**
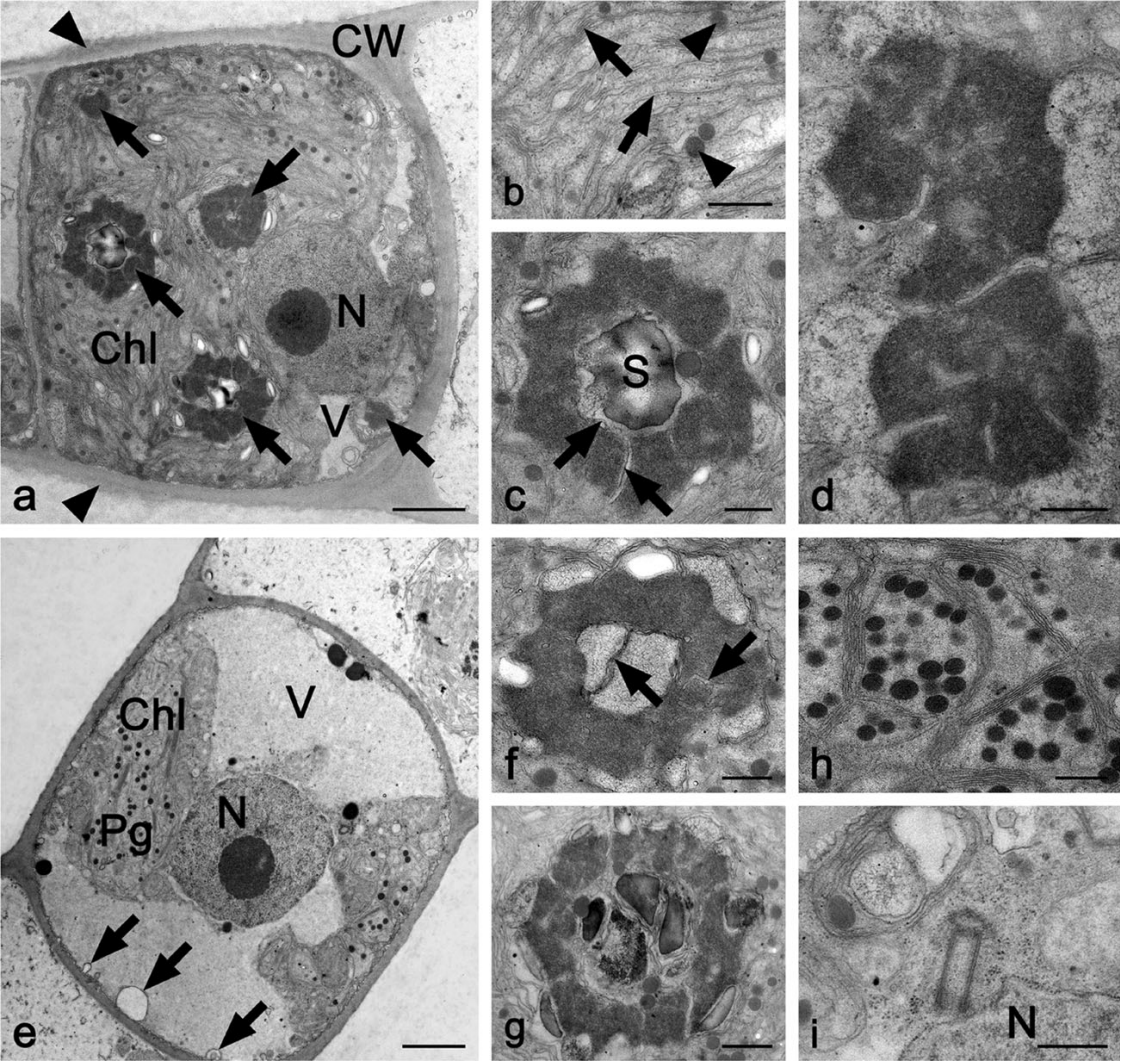
TEM micrographs of longitudinal sections through **a**–**d** UTEX2353 (*Entransia fimbriata*) and **e**–**i** UTEX2793 (*E. fimbriata*). **a** Cell with numerous pyrenoids (*arrows*), small vacuoles, parietal chloroplast, and nucleus; cell wall covered by thin mucilage layer (*arrowheads*). **b** Detail of chloroplast with thylakoid membranes (*arrows*) and plastoglobules (*arrowheads*). **c** Pyrenoid with electron-dense matrix penetrated by thylakoid membranes (*arrow*) and one large starch grain in the center. **d** Two pyrenoids. **e** Cell showing one large vacuole, parietal chloroplast, and central nucleus. Retracted cytoplasm from the cross-wall forms spherical invagination (*arrows*). **f** Pyrenoid with thylakoid membranes in the center (*arrows*) and surrounded by few starch grains. **g** Pyrenoid with starch grains in the center. **h** Detail of chloroplast with numerous plastoglobules. **i** Two centrioles close to the nucleus. *Chl* chloroplast, *CW* cell wall, *N* nucleus, *Pg* plastoglobules, *S* starch grain, *V* vacuole. *Bars* **a**, **e** 2 μm; **b**–**d**, **f**–**i** 500 nm

*E. fimbriata* (UTEX2353) cells contained one strongly expanded parietal chloroplast with thylakoid membranes arranged in parallel, numerous pyrenoids and plastoglobules, a few small vacuoles in the periphery, and one nucleus (Fig. 2a, b). The pyrenoids were usually surrounded by starch grains and differed in their ultrastructure (Fig. 2c, d): while some were traversed by thylakoid membranes and contained starch grains (Fig. 2c), others exhibited electron-dense centers and lacked starch grains (Fig. 2a, d). Occasionally, two pyrenoids occurred in close proximity (Fig. 2d). The homogeneous cell wall was ~0.5–0.7 μm thick, and occasionally a less electron-dense mucilage layer (thickness ~0.7 μm) was attached on the outside of the cell wall (Fig. 2a). The parietal chloroplast of UTEX2793 was less expanded (Fig. 2e). The pyrenoids (at least two per chloroplast) were surrounded by starch grains, penetrated by thylakoid membranes, and either contained or lacked starch grains in the center (Fig. 2f, g). The chloroplast contained thylakoid membranes arranged in parallel and numerous grouped plastoglobules (Fig. 2h). Occasionally, a pair of centrioles occurred near the centrally located nucleus (Fig. 2i). The cell wall was ~0.3–0.6 μm thick, and no outer mucilage layer was observed (Fig. 2e).

**Fig. 3:**
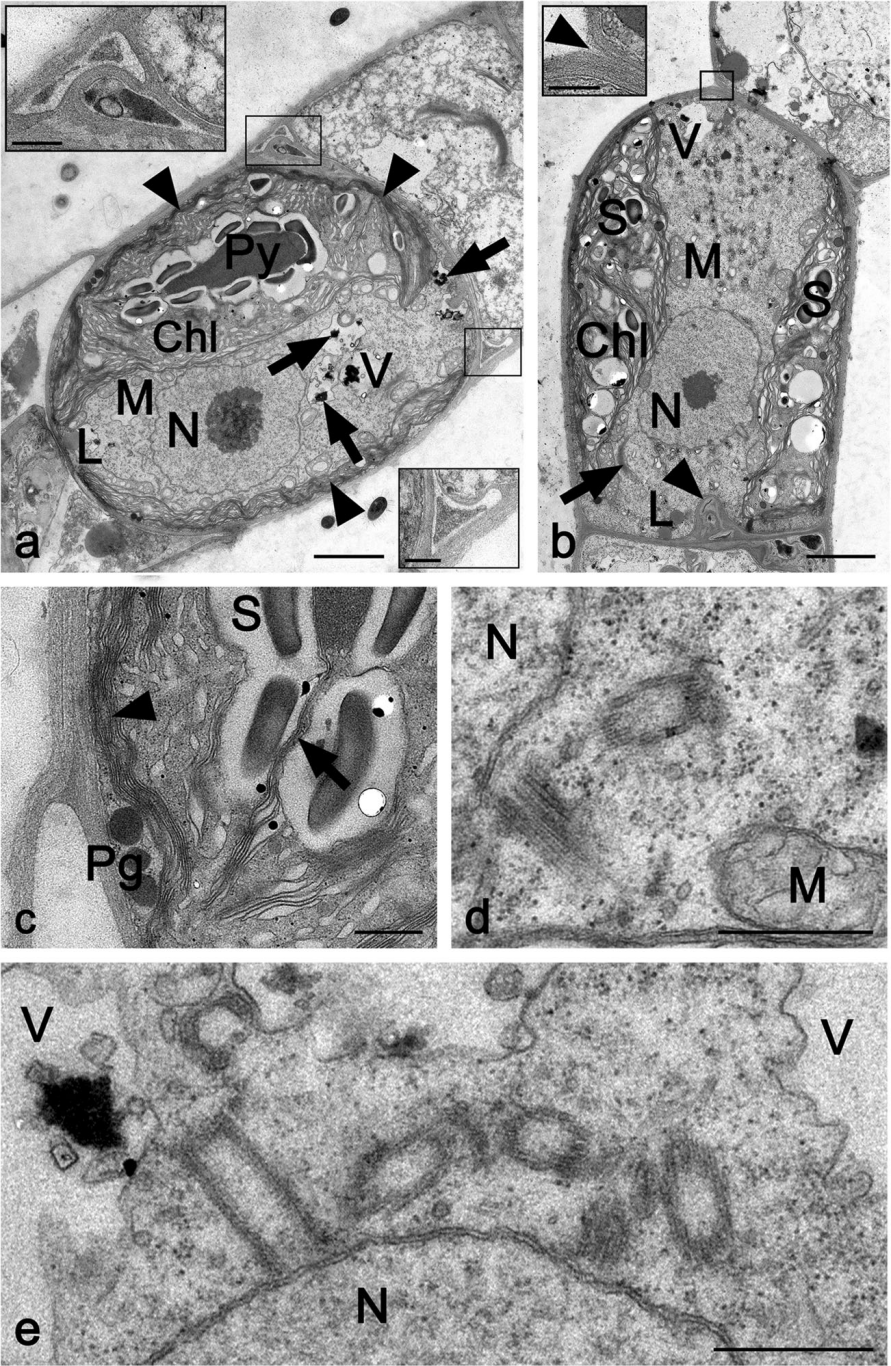
TEM micrographs of longitudinal sections through CCAP329/1 (*Hormidiella attenuata*). **a** Spherical cell containing one parietal chloroplast, with prominent thylakoid layers in the cell periphery (*arrowheads*), one electron-dense pyrenoid surrounded by starch grains, nucleus, mitochondria, small lipid droplet, and small vacuoles filled with electron-dense particles (*arrows*). *Inserts*: Cell corners with cell wall undulations and triangular space between the outer and cross-walls. **b** Detaching cell containing a parietal chloroplast with numerous starch grains, mitochondria, central nucleus, golgi apparatus (*arrow*), lipid bodies, and cross-wall protuberance (*arrowhead*). *Insert*: Detail of detaching cross-wall (*arrowhead*). **c** Detail of chloroplast with starch grains penetrated by thylakoids (*arrow*), stacks of thylakoid membranes near the cell wall (*arrowhead*), and plastoglobules. **d** Pair of centrioles near the nucleus flanked by mitochondrion. **e** Two pairs of periclinally arranged centrioles near the nucleus; vacuoles with or without electron-dense inclusions. *Chl* chloroplast, *CW* cell wall, *L* lipid bodies, *M* mitochondrion, *N* nucleus, *Pg* plastoglobules, *Py* pyrenoids, *S* starch grains, *V* vacuole. *Bars* **a**, **b** 2 μm; **c**–**e**, inserts 500 nm

*H. attenuata* cells (CCAP329/1) contained one large parietal chloroplast with a single homogeneous pyrenoid surrounded by one layer of starch grains (Fig. 3a, b). Parallel thylakoids penetrated the starch layer but not the pyrenoid (Fig. 3c). Numerous thylakoids arranged in parallel were located in close proximity to the plasma membrane, closely attached to the layered cell wall, which was ~0.2–0.5 μm thick (Fig. 3a–c). The cells contained several mitochondria of varying sizes (diameter ~0.3–1.5 μm) near the chloroplast, small vacuoles with electron-dense inclusions, a nucleus, numerous golgi bodies, and a few small, smooth-appearing lipid droplets in the periphery of the cytoplasm (Fig. 3b). Occasionally, one or two pairs of centrioles were found near the periclinal side of the nucleus in vegetative cells (Fig. 3d, e). The cells appeared cylindrical or spherical; the latter were often flanked by cells with unpreserved ultrastructure (i.e., dead when fixed; Fig. 3a). Sometimes, triangular spaces occurred between the outer and cross-walls of a cell (Fig. 3a), and these spaces were filled with inclusions of varying electron density or undulating cell wall material (Fig. 3a). Cell detachment was observed frequently, and the terminal cells became acuminate when the cross-walls detached (Fig. 3b). Cross-wall protuberances were also found (Fig. 3b).

### Light dependence of photosynthesis and respiration (PI-curves)

**Fig. 4:**
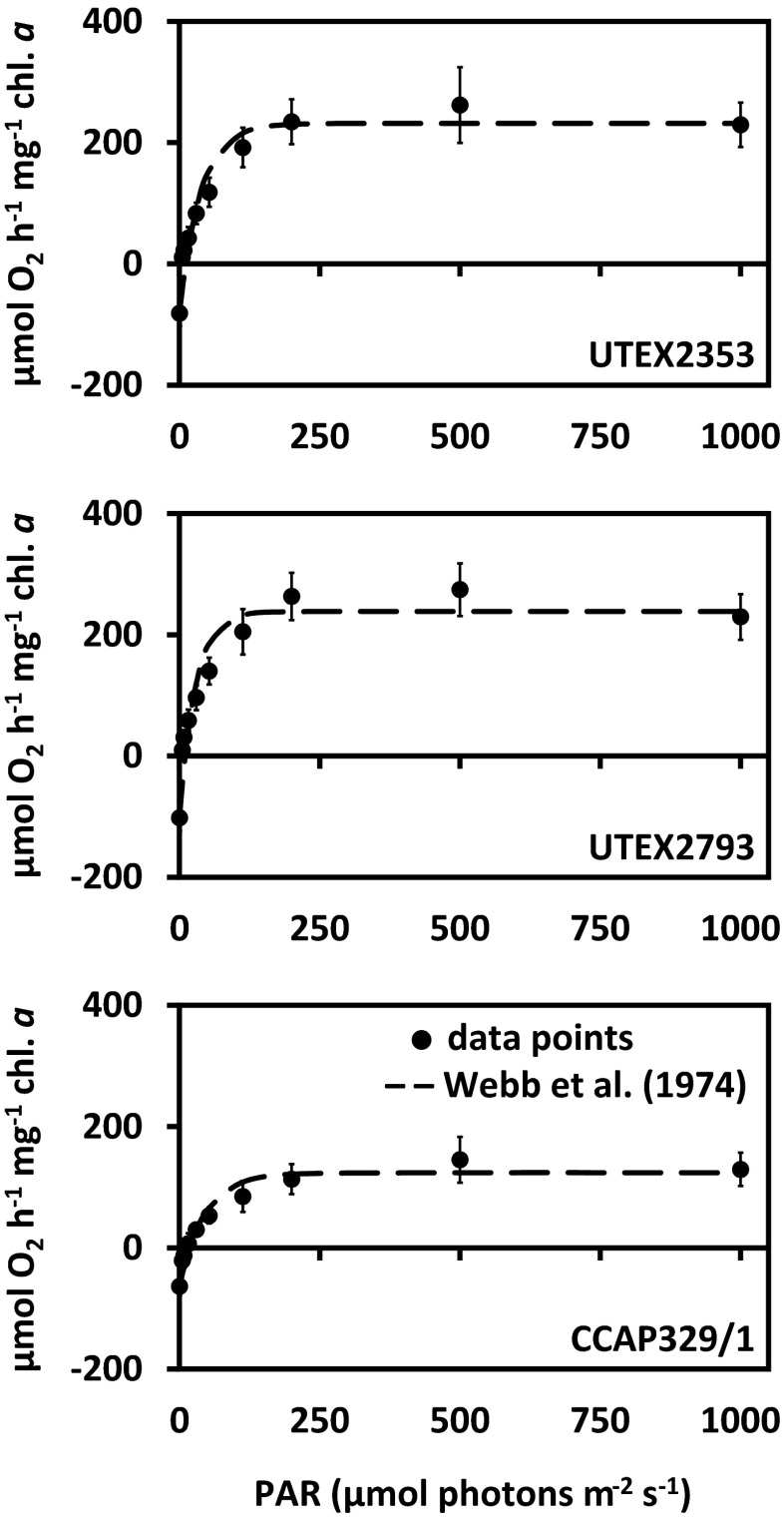
Photosynthetic oxygen production and respiratory consumption in response to increasing PAR up to 1000 μmol photons m^−2^ s^−1^ (PI curves, *n* = 3 ± SD). UTEX2353: *Entransia fimbriata*; UTEX2793: *E. fimbriata*; CCAP329/1: *Hormidiella attenuata*. Data points were fitted according to Webb et al. (1974)

**Fig. 5:**
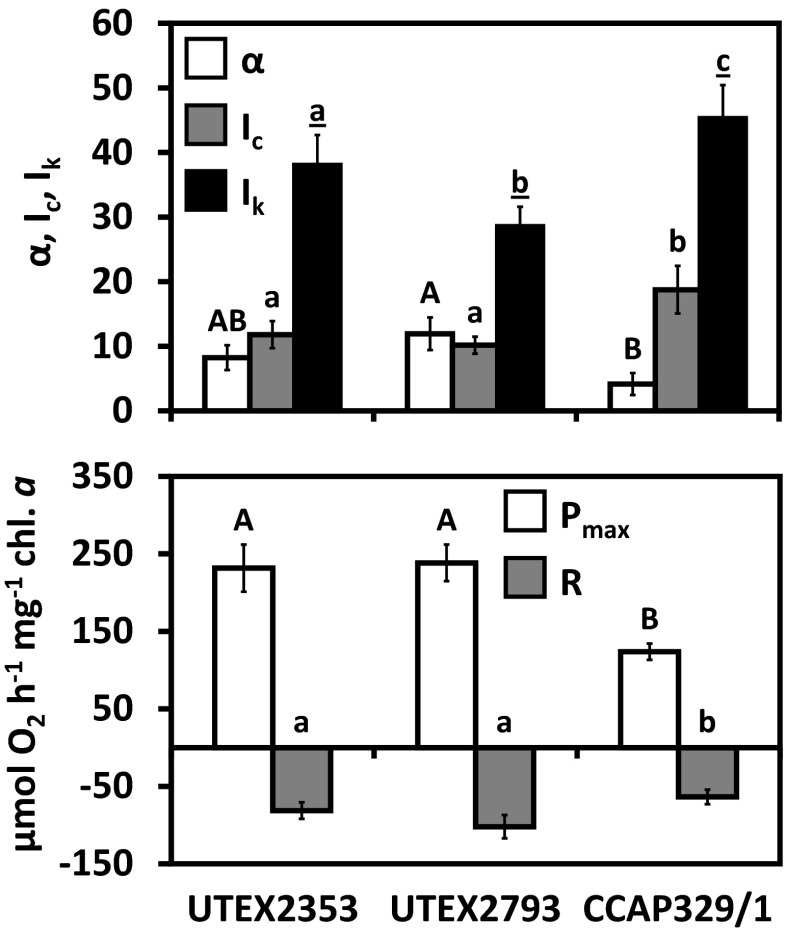
Comparison of five photosynthetic parameters derived from oxygen light curves (Webb et al. 1974) (*n* = 3 ± SD). UTEX2353: *Entransia fimbriata*; UTEX2793: *E. fimbriata*; CCAP329/1: *Hormidiella attenuata*. Significantly different means between the algal strains are indicated by *capital letters* [α; μmol O_2_ h^−1^ mg^−1^ chl. *a* (μmol photons^−1^ m^−2^ s^–1^)^–1^; *P*_max_: μmol O_2_ h^−1^ mg^−1^ chl. *a*], *small letters* (*I*_c_: μmol photons m^−2^ s^−1^; *R*: μmol O_2_ h^−1^ mg^−1^ chl. *a*), and *underlined small letters* (*I*_k_: μmol photons m^−2^ s^−1^). Comparison was performed by one-way ANOVA followed by Tukey’s post hoc test (*P* < 0.05)

**Fig. 6:**
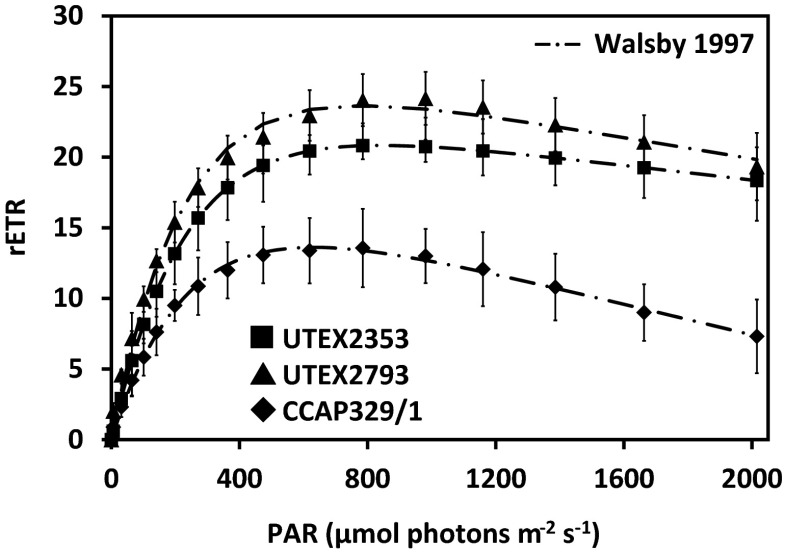
Relative electron transport rate (rETR) curves in response to increasing PAR up to 2015 μmol photons m^−2^ s^−1^ (*n* = 4 ± SD). UTEX2353: *Entransia fimbriata*; UTEX2793: *E. fimbriata*; CCAP329/1: *Hormidiella attenuata*. Data points were fitted according to Walsby (1997)

**Fig. 7:**
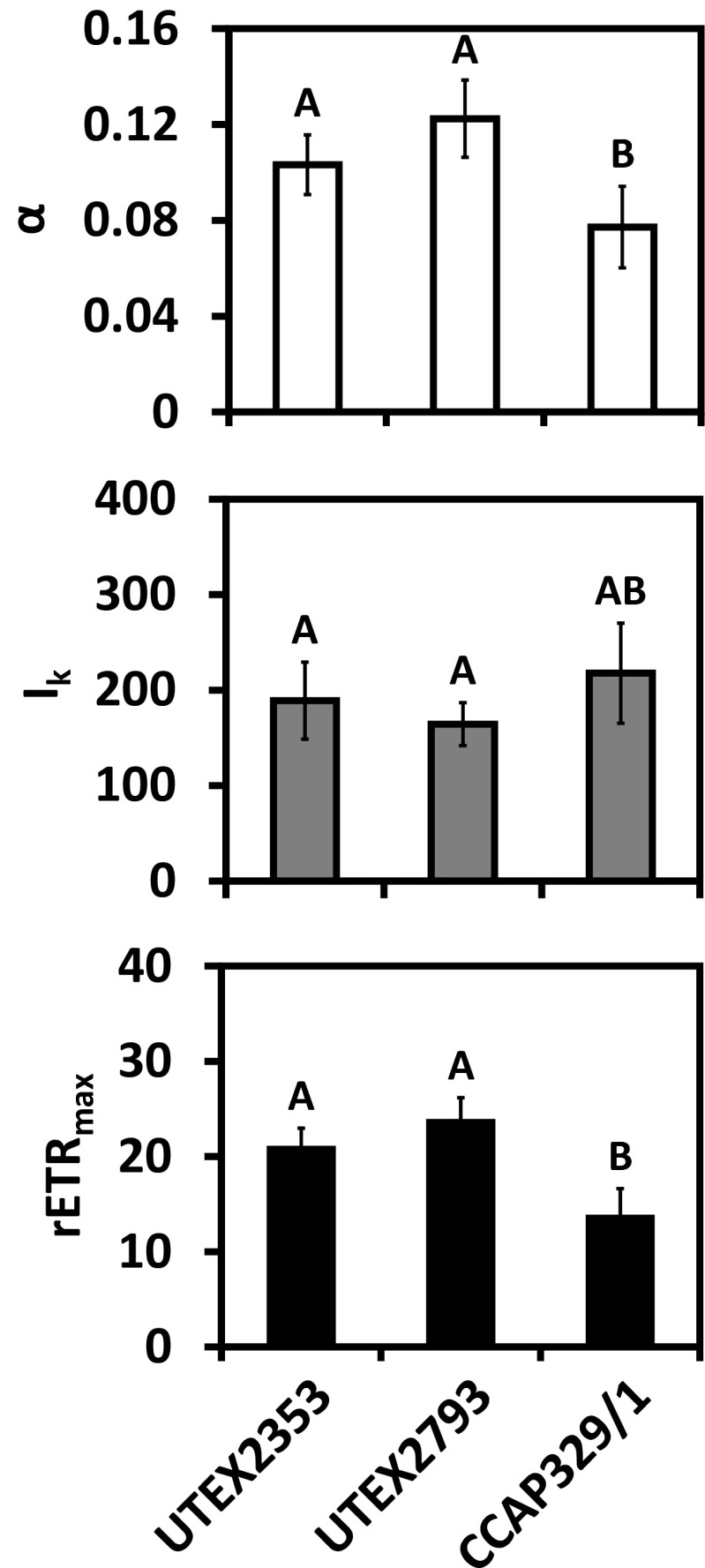
Comparison of the values for α, *I*_k_ (μmol photons m^−2^ s^−1^) and rETR_max_ of UTEX2353 (*Entransia fimbriata*), UTEX2793 (*E. fimbriata*), and CCAP329/1 (*Hormidiella attenuata*) derived from rETR curves according to Walsby (1997) (*n* = 4 ± SD). Significantly different means between the algal strains are indicated by *capital letters*. The values were compared by one-way ANOVA followed by Tukey’s post hoc test (*P* < 0.05)

**Fig. 8:**
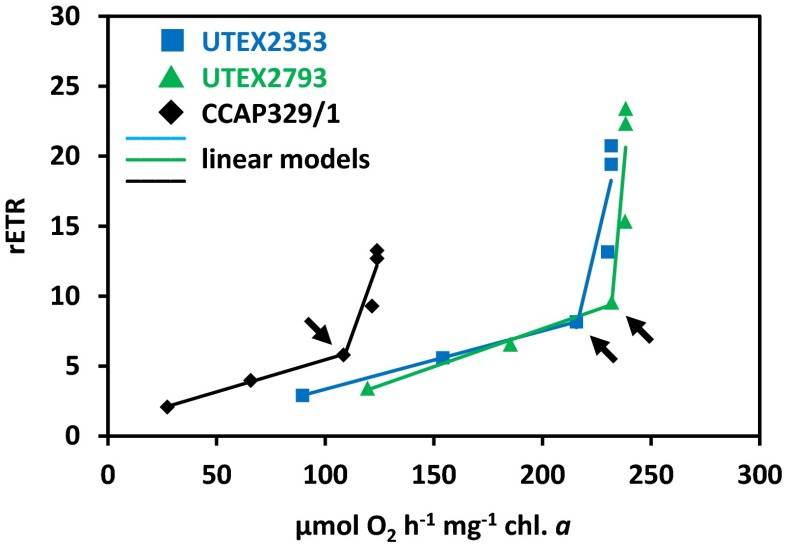
Correlation of the oxygen production and relative electron transport rate (rETR) of UTEX2353 (*Entransia fimbriata*), UTEX2793 (*E. fimbriata*), and CCAP329/1 (*Hormidiella attenuata*) in response to increasing photon fluence rates (30, 55, 105, 200, 490, and 1000 μmol photons m^−2^ s^−1^). Linear regression models were used to describe the correlation from 30 to 105 and from 105 to 1000 μmol photons m^−2^ s^−1^. The oxygen production versus rETR at 105 μmol photons m^−2^ s^−1^ is marked with *arrows*

Photosynthetic oxygen production and respiratory consumption in response to increasing photon fluence rates up to 1000 μmol photons m^−2^ s^−1^, and five calculated photosynthetic parameters revealed sharp differences between the genera (*Entransia*, *Hormidiella*), while the two *Entransia* strains (UTEX2353, UTEX2793) performed similarly (Figs. 4 and 5, Supplementary Table S1). UTEX2793 showed the highest α value, followed by UTEX2353 and CCAP329/1 (Fig. 5, Supplementary Table S1). The light compensation points (*I*_c_) and initial value of light saturation (*I*_k_) of both *Entransia* strains were significantly (*P* < 0.05) lower compared to CCAP329/1 (Fig. 5, Supplementary Table S1). In contrast, both UTEX2353 and UTEX2353 showed a significantly (*P* < 0.05) higher maximum photosynthetic performance (*P*_max_) and respiration (*R*) compared to CCAP329/1. None of the strains showed photoinhibition in response to PAR up to 1000 μmol photons m^−2^ s^−1^ (Fig. 4). Measured rETR as a function of increasing photon fluence rates up to 2015 μmol photons m^−2^ s^−1^, and three derived photosynthetic parameters differed between *Entransia* and *Hormidiella* (Figs. 6 and 7, Supplementary Table S2). The α, *I*_k_, and rETR_max_ values showed no significant differences (*P* < 0.05) in UTEX2353 and UTEX2793, while the α and rETR_max_ values were significantly lower and the *I*_k_ value significantly higher in CCAP329/1 (Fig. 7, Supplementary Table S2). Photoinhibition in response to PAR up to 2015 μmol photons m^−2^ s^−1^ was weak in both *Entransia* strains (UTEX2353, UTEX2793) but increased in CCAP329/1 (Fig. 6). Comparing O_2_ production and rETR kinetics in response to increasing photon fluence rates in UTEX2353, UTEX2793, and CCAP329/1 showed a strong linear correlation from 30 to 105 μmol photons m^−2^ s^−1^ PAR (Fig. 8). This was followed by another linear correlation from 105 to 1000 μmol photons m^−2^ s^−1^ PAR in all three strains (Fig. 8).

### Temperature dependence of photosynthesis and respiration

**Fig. 9:**
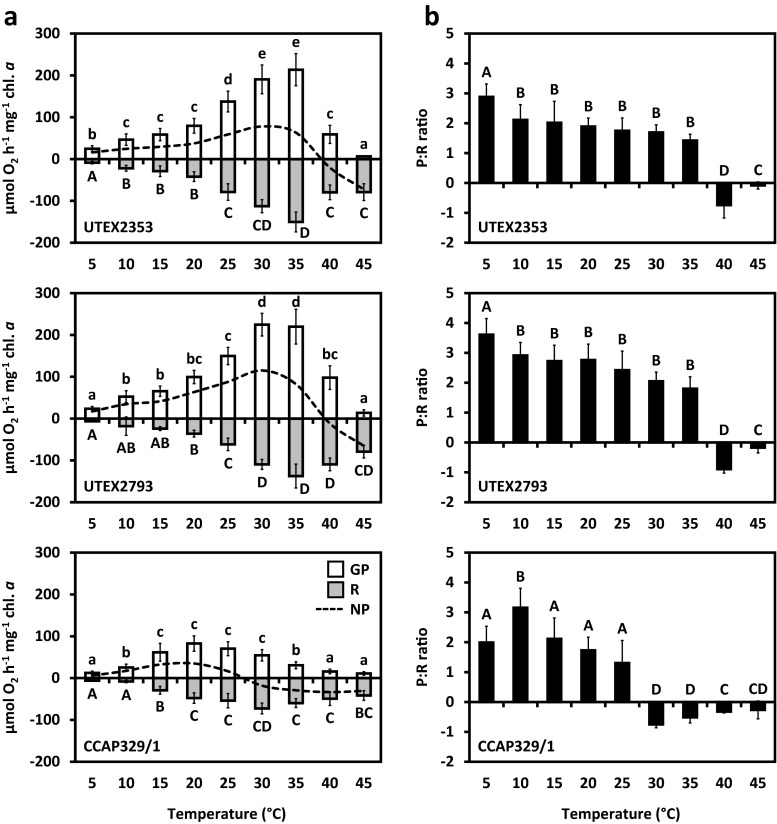
Effect of increasing temperatures (0–45 °C) on **a** gross and net photosynthetic oxygen production and respiratory consumption, and **b** the gross photosynthesis:respiration (P:R) ratio of UTEX2353 (*Entransia fimbriata*), UTEX2793 (*E. fimbriata*), and CCAP329/1 (*Hormidiella attenuata*) (*n* = 3 ± SD; SD values for net photosynthesis are provided in Supplementary Table S3). Significant differences between the temperature steps (*capital letters*: respiration, P:R ratio; *small letters*: gross photosynthesis) were determined by one-way ANOVA followed by Tukey’s post hoc test (*P* < 0.05)

The three strains exhibited strongly temperature-dependent photosynthetic oxygen production and respiratory consumption, and therefore had different temperature requirements for these physiological processes (Fig. 9a). In both *Entransia* strains, gross O_2_ production increased almost linearly from 5 to 35 °C, followed by a sharp decrease at 40 °C, reaching a minimum at 45 °C (Fig. 9a, Supplementary Table S3). Respiration was very low in both *Entransia* strains at low temperatures, and started to increase significantly from 10 °C, before reaching a maximum at 35 °C in UTEX2353 and 30 °C in UTEX2793 (Fig. 9a, Supplementary Table S3). In UTEX2353, respiration decreased again at 40 °C, while in UTEX2793 maximal respiration was maintained up to 40 °C and decreased significantly (*P* < 0.05) at 45 °C (Fig. 9a, Supplementary Table S3). Positive net photosynthesis was measurable at 5 °C in both UTEX2353 and UTEX2793 and increased continuously before reaching a maximum at 30 °C (Fig. 9a, Supplementary Table S1). This was followed by a significant (*P* < 0.05) continuous decrease from 35 to 45 °C, while no positive net photosynthesis occurred at 40 and 45 °C in the two strains respectively (Fig. 9a, Supplementary Table S3). The response of CCAP329/1 to increasing temperatures differed strongly from both *Entransia* strains. Gross photosynthesis increased significantly (*P* < 0.05) from 5 to 10 °C and reached a maximum over a broad range between 15 and 30 °C, followed by a significant decrease at 35 and 40 °C (Fig. 9a, Supplementary Table S3). Respiration increased significantly and almost linearly from 10 to the maximum at 30 °C and was reduced significantly at 35 and 45 °C (Fig. 9a, Supplementary Table S3). Positive net photosynthesis occurred between 5 and 25 °C with a maximum at 20 °C (Fig. 9a, Supplementary Table S3). In both UTEX 2353 and UTEX2793, the gross photosynthesis:respiration (P:R) was highest at 5 °C (2.88 ± 0.43 and 3.61 ± 0.54), decreased significantly (*P* < 0.05) at 10 °C, and remained unchanged till 35 °C, followed by a strong drop to negative values at 40 and 45 °C (Fig. 9b). In contrast, in CCAP329/1, the highest P:R ratio was measured at 10 °C (3.16 ± 0.65; Fig. 9b); P:R ratios were significantly lower (*P* < 0.05) at 5 and 15–20 °C and negative between 30 and 45 °C (−0.75 ± 0.11 to −0.26 ± 0.30, Fig. 9b).

### Desiccation experiment

**Fig. 10:**
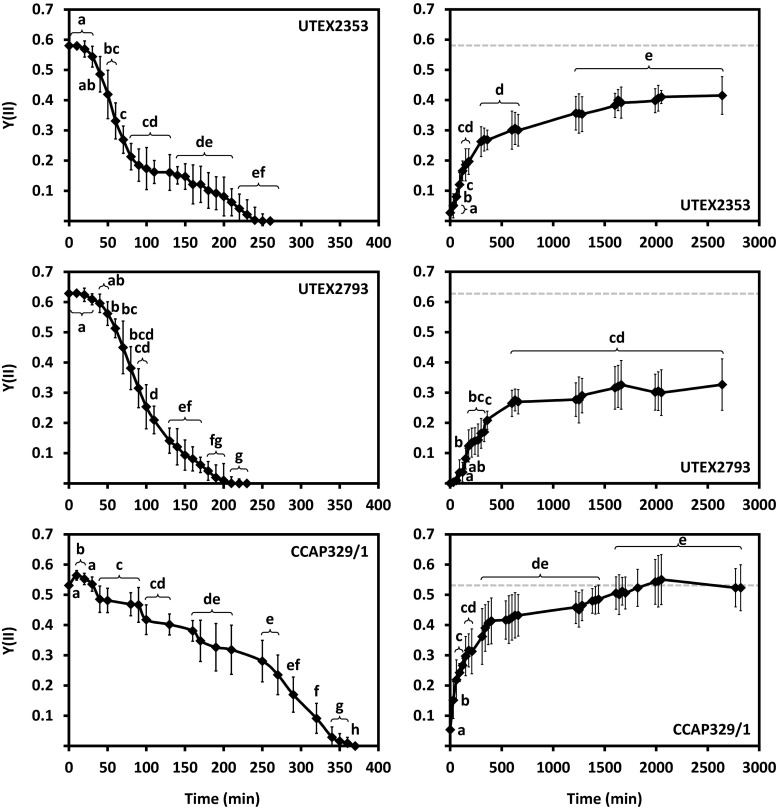
Effect of desiccation at ~10 % RH (*left*) followed by rehydration (*right*) on the effective quantum yield of PSII [Y(II)] of UTEX2353 (*Entransia fimbriata*), UTEX2793 (*E. fimbriata*), and CCAP329/1 (*Hormidiella attenuata*) (*n* = 4 ± SD). The *dashed gray lines* on the rehydration charts indicate the control Y(II) value. During the experiment, algae were exposed to 40 μmol photons m^−2^ s^−1^ at 22 ± 0.5 °C. Significant differences between the groups, determined by one-way ANOVA (*P* < 0.05) followed by Tukey’s post hoc test, are indicated by *small letters*

Desiccation over silica gel (RH ~ 10 %) sharply decreased the Y(II) of UTEX2353 and UTEX2793, while the two strains showed similar kinetics (Fig. 10). In UTEX2353, the Y(II) of control samples (0.58 ± 0.02) decreased significantly after 40 min, dropped sharply to ~0.2 after 80 min of desiccation, and then remained unchanged for 40 min (Fig. 10). This was followed by an almost linear decrease until no Y(II) was measurable after 210 min exposure (Fig. 10). After rehydration, the Y(II) started to recover immediately and reached ~45 % of the control value after 5 h (Fig. 10). Forty-four hours of rehydration allowed the Y(II) to recover to ~75 % of the initial value (Fig. 10). The control Y(II) of UTEX2793 (0.628 ± 0.01) was higher than the value of UTEX2353, but decreased similarly after 40 min of desiccation (Fig. 10). This was followed by a linear decrease of Y(II), reaching 0 after 230 min treatment (Fig. 10). Recovery of the Y(II) in UTEX2793 started after 60 min of rehydration (Fig. 10). After 6 h, ~33 % of the control value was reached (Fig. 10) and after 44 h of rehydration, ~50 % of the initial Y(II) was restored (Fig. 10). The Y(II) of CCAP329/1 was much less sensitive to reduced water availability compared to both UTEX2353 and UTEX2793, and 10 min of desiccation even led to a significant (*P* < 0.05) increase of the control value (from 0.53 ± 0.01 to 0.57 ± 0.02; Fig. 10). After 30 min, the Y(II) decreased almost linearly and reached 0 after 370 min of desiccation (Fig. 10). Immediately upon rehydration, the Y(II) began to recover, and after 5 h was ~75 % restored (Fig. 10). Full recovery was measured after ~24 h (Fig. 10).

## Discussion

Similarly to several species of closely related *Klebsormidium* and *Interfilum*, *H. attenuata* occurs in habitats with low water availability (Lokhorst et al. 2000; Mikhailyuk et al. 2008; Holzinger and Karsten 2013). In contrast, *E. fimbriata* prefers limnetic transition zones (Cook 2004), where water availability is not the key ecological factor. However, as in many soils, these humid habitats frequently undergo fluctuating light and temperature regimes (Finlay et al. 2001; Schubert et al. 2001). In order to remain metabolically active in such rapidly changing environments, it is crucial for algae to maintain sufficient photosynthetic performance under a broad range of photon fluence rates and temperatures.

### Light requirements

We found that both *E. fimbriata* strains investigated (UTEX2353, UTEX2793) used light more efficiently for photosynthesis than did *H. attenuata* (CCAP329/1). This was indicated by higher *P*_max_ and rETR_max_ values derived from light response curves. Both UTEX2353 and UTEX2793 were only slightly photoinhibited under high light conditions, while in CCAP329/1, the rETR at the highest photon fluence rate (PAR 2015 μmol photons m^−2^ s^−1^) was ~45 % lower compared to rETR_max_. Furthermore, *Entransia* showed a higher photosynthetic efficiency under low light conditions (α) and lower light compensation (I_c_) and saturation (I_k_) points. Interestingly, the highest α and lowest *I*_k_ values were measured in UTEX2793, which was obtained from a *Sphagnum* bog (Cook 2004). The accumulated peat, humic particles, and dissolved organic carbon (DOC) decrease the water transparency in these mires (Belyea and Warner 1996), reducing light penetration and drastically decreasing the depth of the euphotic zone (Eloranta 1978). This explains the distinct low-light adaptation of UTEX2793. In addition, UTEX2353 was collected from the littoral zone of Fawn Lake (Canada; Cook 2004), which differs from other soft-water lakes in the area in its humic-stained water and significantly higher DOC fraction (West et al. 2003). Thus, algae occurring in Fawn Lake are also frequently exposed to low light conditions, as they are submersed in water enriched with shading particles. As reported recently, both UTEX2353 and UTEX2793 showed strong photoinhibition when exposed to ultraviolet radiation (UVR), due to the lack of MAAs (Kitzing and Karsten 2015). In *Klebsormidium* and *Hormidiella*, MAAs reduce harmful UVR effects on the photosynthetic apparatus (Kitzing and Karsten 2015). Therefore, the restriction of UTEX2353 and UTEX2793 to dim limnetic habitats with high amounts of strongly UVR-absorbing DOC (Morris et al. 1995; West et al. 2003) might be an adaptation to UVR sensitivity, while their conspicuously low light requirement for photosynthesis allows them to remain metabolically active. In CCAP329/1, the low light requirements were in the range of those of *Interfilum* strains obtained from soil habitats (Karsten et al. 2014). The values for α, *I*_c_, and *I*_k_ derived from oxygen evolution curves in the present study were similar to those measured in *Interfilum massjukiae* (SAG2102), which was obtained from soil covering pyroclastic outcrops in Crimea (Ukraine; Karsten et al. 2014). Interestingly, the maximum photosynthetic O_2_ production of CCAP329/1 was in the range of several *Klebsormidium* species that also occur in soil habitats (Kaplan et al. 2012; Karsten and Holzinger 2012; Karsten et al. 2013), while UTEX2353 and UTEX2793 showed a higher maximum O_2_ production, comparable to some species of *Zygnema* (Zygnematophyceae) from limnetic transitional habitats (Kaplan et al. 2013). Thus, the different light responses of *Entransia* and *Hormidiella* concord with their different natural habitats. However, the three strains also had some responses in common. Similar to several green algae belonging to the Klebsormidiophyceae (Kaplan et al. 2012; Karsten et al. 2013, 2014) and Zygnematophyceae (Remias et al. 2012; Aigner et al. 2013; Herburger et al. 2015), all strains investigated in this study showed no photoinhibition under high photon fluence rates in the range up to 500 μmol photons m^−2^ s^−1^. In combination with the lack of photoinhibition, these data indicate a high photophysiological plasticity, which seems to be a common trait in many members of Klebsormidiophyceae (Karsten et al. 2010, 2013, 2014) and Zygnematophyceae (Kaplan et al. 2013; Herburger et al. 2015). Furthermore, in both *Entransia* and *Hormidiella*, *I*_k_ for rETR_max_ was reached under much higher photon fluence rates compared to the corresponding value for *P*_max_ and the multiplication factors between these two values were similar (UTEX2353: 5.2, UTEX2793: 5.6, CCAP329/1: 4.8). Similar factors were found in two species of *Zygnema* (Zygnematophyceae) obtained from hydroterrestrial habitats (Herburger et al. 2015). This is also reflected by correlating the rETR with the oxygen production at increasing photon fluence rates, which resulted in a positive linear increase from ~30 to ~100, and another one from ~100 to ~1000 μmol photons m^−2^ s^−1^. Interestingly, in all three strains investigated here, a much higher increase occurred above ~100 μmol photons m^−2^ s^−1^, indicating decreasing oxygen production in relation to electron transport through PSII. A linear correlation between O_2_ production and electron transport at lower photon fluence rates was previously reported in several species of marine algae (Longstaff et al. 2002; Carr and Björk 2003; Beer and Axelsson 2004). As assumed for tropical seagrasses, decreasing oxygen production in relation to electron flow in *Entransia* and *Hormidiella* might be attributed to an increase in photorespiration (Beer and Björk 2000), cyclic electron flow at PSII (Falkowski et al. 1986), or the Mehler reaction (Asada 2000) at high irradiances.

### Desiccation tolerance

CCAP329/1 was isolated originally from soil in a xeromorphic forest (Lokhorst et al. 2000). The amount of annual rainfall in this region is similar to the limnetic habitats of both strains of *Entransia*, and much higher than for the soil habitat of *I. massjukiae* SAG2102 (Karsten et al. 2014). However, precipitation at the collection site of CCAP329/1 is very low in the spring and summer months (May to Aug; monthly precipitation 31–62 mm). Because of the soil habitat and these extended dry periods during the year, CCAP329/1 is more frequently exposed to desiccation stress than are the *Entransia* strains investigated. Both UTEX2353 and UTEX2793 failed to show measurable photosynthesis after ~3.5 h, while the Y(II) of CCAP329/1 still amounted to ~50 % of the initial value, and full recovery was possible after rehydration. Thus, CCAP329/1 might possess desiccation-tolerance mechanisms that are similar to those of *Interfilum* and other aero-terrestrial green algae, which showed similar physiological responses under conditions of low water availability (Häubner et al. 2006; Karsten et al. 2014). In contrast, the photosynthetic performance of green algae from limnetic habitats is strongly inhibited under desiccation stress, and does not recover fully upon rehydration (Holzinger and Karsten 2013). However, compared to other freshwater green algae (Gray et al. 2007; Herburger et al. 2015), both *Entransia* strains investigated in this study were less affected by desiccation stress. Withstanding dehydration to some extent might be beneficial for organisms living in the littoral zone of lakes or in bogs, where the microtopography consisting of hummocks and water-filled hollows (Schipperges and Rydin 1998) leads to frequent changes in water availability, due to evaporation and changes in the level of the groundwater (Rydin 1985; Lafleur et al. 2005).

### Temperature dependence

Similarly to other green algae belonging to the Klebsormidiophyceae (Karsten et al. 2010, 2014; Karsten and Holzinger 2012) and Zygnematophyceae (Remias et al. 2012; Herburger et al. 2015), in both *Entransia* and *Hormidiella*, photosynthetic oxygen production and respiratory consumption depended strongly on temperature. In general, the light reaction of photosynthesis is less temperature-dependent than respiration, which occurs through an enzyme chain with different temperature optima. A temperature decrease to below the operating temperature of only one enzymatic process can act as a bottleneck and restrict respiration (Atkin and Tjoelker 2003). In both *Entransia* strains, positive net photosynthesis was measured from 5 to 35 °C, with an optimum between 25 and 35 °C. However, the highest gross photosynthesis:respiration (P:R) ratios occurred at 5 °C, indicating a high net carbon gain (i.e., biomass formation) at lower temperatures. This reflects the natural habitats of both *Entransia* strains, in which they are usually submersed in lake or bog water with a maximum air temperature not exceeding 25 °C. In contrast, positive net photosynthesis in CCAP329/1 occurred in a narrower range (5–25 °C), and the highest P:R ratio was measured at 10 °C. The annual mean temperature of the region where CCAP329/1 was obtained is 18.3 °C (www.worldclimate.com), and the warmest months (Jan–Mar and Oct–Dec) coincide with the highest precipitation. Biomass formation likely occurs during these months, as soon as sufficient rainwater is available, which cools the soil habitat of CCAP329/1 and provides suitable growing temperatures.

### Structural adaptation to the environment

The ultrastructure of CCAP329/1 has been described by Lokhorst et al. (2000), and their basic findings were confirmed in this study. However, Lokhorst et al. (2000) did not describe two pairs of centrioles located on the periclinal side of the nucleus of some vegetative cells. The appearance of these centrioles might be a precursor to cell division, as they shift to the cell poles during mitosis and likely help to separate the chromosomes (Lokhorst et al. 2000). Furthermore, we found triangular spaces between the longitudinal and cross walls of cells, and these spaces were filled with material of varying electron density or undulating cell walls. Similar local modifications of the cell wall are found in *Entransia* (Cook 2004), *Interfilum*, and *Klebsormidium* (Mikhailyuk et al. 2014). In *Klebsormidium*, they usually contain high amounts of callose and likely contribute to cell wall plasticity (Holzinger et al. 2011, Herburger and Holzinger 2015), which is important for a regulated shrinkage and expansion of the cells during cellular water loss and rehydration, respectively. Cross-wall protuberances were found frequently in CCAP329/1. These structures also occur in *Entransia* (Cook 2004), and might be related to cell detachment. The *Hormidiella* strain exhibited a much stronger tendency to fragment compared to *Entransia*. As shown by transmission electron microscopy (TEM), the integrity of the basic organelles (chloroplast, mitochondria, nucleus) in most terminal cells of fragments remained intact. Therefore, fragmentation into short filaments might be a rapid and economical means of producing undamaged dispersal units under changing environments (i.e., climate change), as short cell filaments can be dispersed by atmospheric transport (Sharma et al. 2007). Although information on the biogeographical distribution of *Hormidiella* is very limited, it is likely to occur worldwide, as the spore formation, the capability to form short cell filaments, and the cell dimensions are similar to the cosmopolitan genus *Klebsormidium* (Lokhorst 1996; Lokhorst et al. 2000; Škaloud and Rindi 2013). Furthermore, light microscopy as well as TEM revealed that the terminal cells of *Hormidiella* filaments tended to narrow toward the polar ends. This might be a consequence of the detachment process, which shapes the terminal cross-wall. Acuminate terminal cells might aid individual algal filaments in penetrating the uppermost biological soil layers to form tightly woven mats. Especially in habitats with frequent low water availability and strong irradiation, formation of these layers on the soil surface is one mechanism to aid green algae in coping with harsh environmental conditions (Karsten and Holzinger 2014).

UTEX2353 and UTEX2793 belong to the same species (*E. fimbriata*), as they are similar on the DNA level (Cook 2004; Sluiman et al. 2008). However, certain morphological and ultrastructural differences between these two strains appeared. (The pair of centrioles found near the nucleus in a few vegetative UTEX2793 cells may not constitute such a difference, since it is likely also detectable in UTEX2353). UTEX2353 contained a more-developed chloroplast, which extended to the cross-wall; while the degree of vacuolization was higher in UTEX2793. Furthermore, the cell wall of UTEX2353 was slightly thicker compared to UTEX2793 and was occasionally covered with an outer mucilage layer. In cyanobacteria and green algae, such amorphous layers of extracellular polysaccharides restrict cellular water loss and support photosynthetic performance during desiccation stress (Tamaru et al. 2005; Karsten et al. 2014). As mucilage layers were seldom found in UTEX2353, they only partly explain this strain’s slightly higher tolerance to short-term desiccation stress compared to UTEX2793. The different degrees of vacuolization of UTEX2353 (low) and UTEX2793 (high) might also play a role. Finally, the different responses to desiccation might be related to slightly different water regimes in their natural habitats, and imply a high phenotypic plasticity, which is a common phenomenon in green algae (Lürling 2003).

Interestingly, this ability of *E. fimbriata* to produce more than one morphological form and physiological behavior in response to different habitats is very stable, since both strains investigated in this study had the same culture age (1 month) and were obtained from long-term culture collections. A physiological performance and ultrastructure closely related to the original habitat were also found in the green algae *Cosmarium* (Stamenković and Hanelt 2013; Stamenković et al. 2014) and *Zygnema* (Herburger et al. 2015) after long-term culture. Furthermore, Cook (2004) found that the morphology of cultured *Entransia* filaments appeared very similar to field specimens.

## Conclusion

The photosynthetic responses of *E. fimbriata* (UTEX2353, UTEX2793) and *H. attenuata* (CCAP329/1) to light and temperature gradients and desiccation stress differed strongly, and were related to the different habitats where the algae had been collected. Both *Entransia* strains are well adapted to dim humid environments, while CCAP329/1 showed a higher tolerance to desiccation stress, consonant with its preference for soil habitats. Although UTEX2353 and UTEX2793 represent the same species, their photosynthetic response patterns to abiotic stress, as well as their morphology and ultrastructure differed to some extent, indicating a high phenotypic plasticity of *Entransia fimbriata*, which was maintained even after long-term culture. The occurrence of centrioles in vegetative cells of *Entransia* and *Hormidiella* is one difference from *Klebsormidium* and *Interfilum*, which lack these structures.

## Electronic supplementary material

Below is the link to the electronic supplementary material.

Supplementary Table S1 (DOCX 14 kb)

Supplementary Table S2 (DOCX 14 kb)

Supplementary Table S3 (DOCX 17 kb)

Supplementary Fig. S1 Relative air humidity (RAH) and temperature inside the desiccation chamber during the experiment with CCAP329/1 (Hormidiella attenuata). After an adjustment period of 100 min, algae were placed in the chamber containing silica gel (100 g) and desiccation measurements were started. Immediately after the desiccation period, the silica gel was replaced with tap water (100 mL) and 200 μL of culture medium (modified BBM) was added to the dried algae for rehydration measurement. (PPTX 76 kb)
