# Supplementary material for: Entransia and Hormidiella, sister lineages of Klebsormidium (Streptophyta), respond differently to light, temperature, and desiccation stress
Source: Protoplasma. 2015 Oct 6;253(5):1309–23. doi: 10.1007/s00709-015-0889-z (PMC4710678; doi:10.1007/s00709-015-0889-z)
Supplement: Supplementary file 1 — (DOCX 14 kb) [file 709_2015_889_MOESM1_ESM.docx]

|  | **α** | **Ic** | **Ik** | **Pmax** | **R** |
| --- | --- | --- | --- | --- | --- |
| **UTEX2353** | 8.24 ± 1.92AB | 11.7 ± 2.10A | 37.99 ± 4.71A | 231.60 ± 30.45A | -81.28 ± 10.66A |
| **UTEX2793** | 11.93 ± 2.53A | 10.17 ± 1.32A | 28.52 ± 3.09B | 238.31 ± 23.62A | -102.05 ± 14.97A |
| **CCAP329/1** | 4.14 ± 1.68B | 18.75 ± 3.69B | 45.24 ± 5.18C | 123.78 ± 10.54B | -63.56 ± 9.43B |

Different capital letters indicate significant differences between the values of UTEX2353 (*Entransia fimbriata*), UTEX2793 (*E. fimbriata*) and CCAP329/1 (*Hormidella attenuata*). They were determined by one-way ANOVA followed by Tukey’s post hoc test (P<0.05). α initial slope in the light-limiting range (μmol O_2_ h^−1^ mg^−1^ chl. *a* (μmol photons m^−2^ s^−1^)^-1^), I_c_ light compensation point (μmol photons m^-2^ s^-1^), I_k_ initial value of light-saturated photosynthesis (μmol photons m^-2^ s^-1^), P_max_ maximum photosynthetic rate in the light-

saturated range (μmol O_2_ h^−1^ mg^−1^ chl. *a*), and R respiration rate in the dark (μmol O_2_ h^−1^ mg^−1^ chl. *a*)
