# Supplementary material for: Entransia and Hormidiella, sister lineages of Klebsormidium (Streptophyta), respond differently to light, temperature, and desiccation stress
Source: Protoplasma. 2015 Oct 6;253(5):1309–23. doi: 10.1007/s00709-015-0889-z (PMC4710678; doi:10.1007/s00709-015-0889-z)
Supplement: Supplementary file 2 — (DOCX 14 kb) [file 709_2015_889_MOESM2_ESM.docx]

|  | **α** | **Ik** | **rETRmax** |
| --- | --- | --- | --- |
| **UTEX2353** | 0.10 ± 0.01A | 189.08 ± 40.33A | 20.83 ± 2.13A |
| **UTEX2793** | 0.12 ± 0.02A | 164.36 ± 22.46A | 23.66 ± 2.51A |
| **CCAP329/1** | 0.07 ± 0.02B | 217.74 ± 52.37AB | 13.61 ± 3.00B |

Different capital letters indicate significant differences between the values of UTEX2353 (*Entransia fimbriata*), UTEX2793 (*E. fimbriata*) and CCAP329/1 (*Hormidella attenuata*). They were determined by one-way ANOVA followed by Tukey’s post hoc test (P<0.05). α initial slope at limiting photofluence rates (electrons photon^−1^), I_k_ initial value of light-saturated photosynthesis (μmol photons m^-2^ s^-1^), rETR_max_ maximum electron transport rate (μmol electrons m^−2^ s^−1^)
