# Supplementary material for: Entransia and Hormidiella, sister lineages of Klebsormidium (Streptophyta), respond differently to light, temperature, and desiccation stress
Source: Protoplasma. 2015 Oct 6;253(5):1309–23. doi: 10.1007/s00709-015-0889-z (PMC4710678; doi:10.1007/s00709-015-0889-z)
Supplement: Supplementary file 3 — (DOCX 17 kb) [file 709_2015_889_MOESM3_ESM.docx]

| **TEMP** |  | **UTEX2353** |  |  |  | **UTEX2793** |  |  |  | **CCAP329/1** |  |
| --- | --- | --- | --- | --- | --- | --- | --- | --- | --- | --- | --- |
| **(°C)** | **GP** | **R** | **NP** |  | **GP** | **R** | **NP** |  | **GP** | **R** | **NP** |
| **5** | 24.66 ± 6.99b | -8.56 ± 3.01A | 16.1 ± 1.28a |  | 23.66 ± 5.39A | -6.56 ± 1.28A | 17.1 ± 3.02a |  | 12.36 ± 4.23a | -6.21 ± -0.77A | 6.15 ± 2.19a |
| **10** | 46.46 ± 13.66c | -22.01 ± 6.99B | 24.46 ± 9.01b |  | 52.46 ± 14.16B | -18.01 ± 22.01A | 34.46 ± 6.99b |  | 25.36 ± 7.96b | -8.04 ± -2.92AA | 17.32 ± 5.27b |
| **15** | 58.46 ± 14.6c | -29.04 ± 12.65B | 29.42 ± 4.36b |  | 65.46 ± 12.24B | -24.04 ± 4.36AB | 41.42 ± 12.65b |  | 61.55 ± 21.37c | -29.14 ± -9.28B | 32.41 ± 6.13c |
| **20** | 79.46 ± 17.57c | -42.05 ± 11.57B | 37.4 ± 7.99Bc |  | 99.46 ± 15.9BC | -36.05 ± 7.99B | 63.4 ± 11.57Bc |  | 82.65 ± 18.37c | -47.75 ± -12.54C | 34.9 ± 4.16c |
| **25** | 137.57 ± 24.76d | -78.77 ± 19.88C | 58.79 ± 14.97c |  | 149.57 ± 20.64C | -61.77 ± 14.97C | 87.79 ± 19.88c |  | 70.34 ± 16.63c | -54.00 ± -17.03C | 16.34 ± 5.35b |
| **30** | 190.57 ± 34.3e | -112.62 ± 15.66CD | 77.94 ± 17.99c |  | 224.57 ± 27.12D | -109.62 ± 12D | 114.94 ± 15.66d |  | 54.51 ± 13.64c | -72.56 ± 12.8CD | -18.04 ± 11.21d |
| **35** | 213.57 ± 38.44e | -150.43 ± 23.66D | 63.13 ± 18.58c |  | 219.72 ± 41.37D | -137.43 ± 28.58D | 82.29 ± 23.51c |  | 30.65 ± 8.24b | -59.87 ± -10.53C | -29.21 ± 9.89d |
| **40** | 59.24 ± 21.65c | -79.53 ± 17.57C | -20.29 ± 15.11d |  | 97.88 ± 28.17BC | -109.53 ± 15.11D | -11.65 ± 17.57e |  | 15.83 ± 5.3a | -49.26 ± -16.08C | -33.44 ± -12.32d |
| **45** | 6.57 ± 4.64a | -79 ± 20C | -72.44 ± 15.01e |  | 13.57 ± 7.64A | -79 ± 15.01CD | -65.44 ± 20.09f |  | 10.99 ± 4.32a | -41.61 ± -11.55BC | -30.61 ± 14.05d |

Significant differences between the temperature steps (small letters: gross photosynthesis (GP: μmol O_2_ h^−1^ mg^−1^ chl. *a*), capital letters: respiration (R: μmol O_2_ h^−1^ mg^−1^ chl. *a*), underlined small letters: nett photosynthesis (NP: μmol O_2_ h^−1^ mg^−1^ chl. *a*) in each strain were determined by one-way ANOVA followed by Tukey’s post hoc test (P<0.05).
