# Supplementary material for: Entransia and Hormidiella, sister lineages of Klebsormidium (Streptophyta), respond differently to light, temperature, and desiccation stress
Source: Protoplasma. 2015 Oct 6;253(5):1309–23. doi: 10.1007/s00709-015-0889-z (PMC4710678; doi:10.1007/s00709-015-0889-z)
Supplement: Supplementary file 4 — Relative air humidity (RAH) and temperature inside the desiccation chamber during the experiment with CCAP329/1 (Hormidiella attenuata). After an adjustment period of 100 min, algae were placed in the chamber containing silica gel (100 g) and desiccation measurements were started. Immediately after the desiccation period, the silica gel was replaced with tap water (100 mL) and 200 μL of culture medium (modified BBM) was added to the dried algae for rehydration measurement. (PPTX 76 kb) [file 709_2015_889_MOESM4_ESM.pptx]

## Slide 1
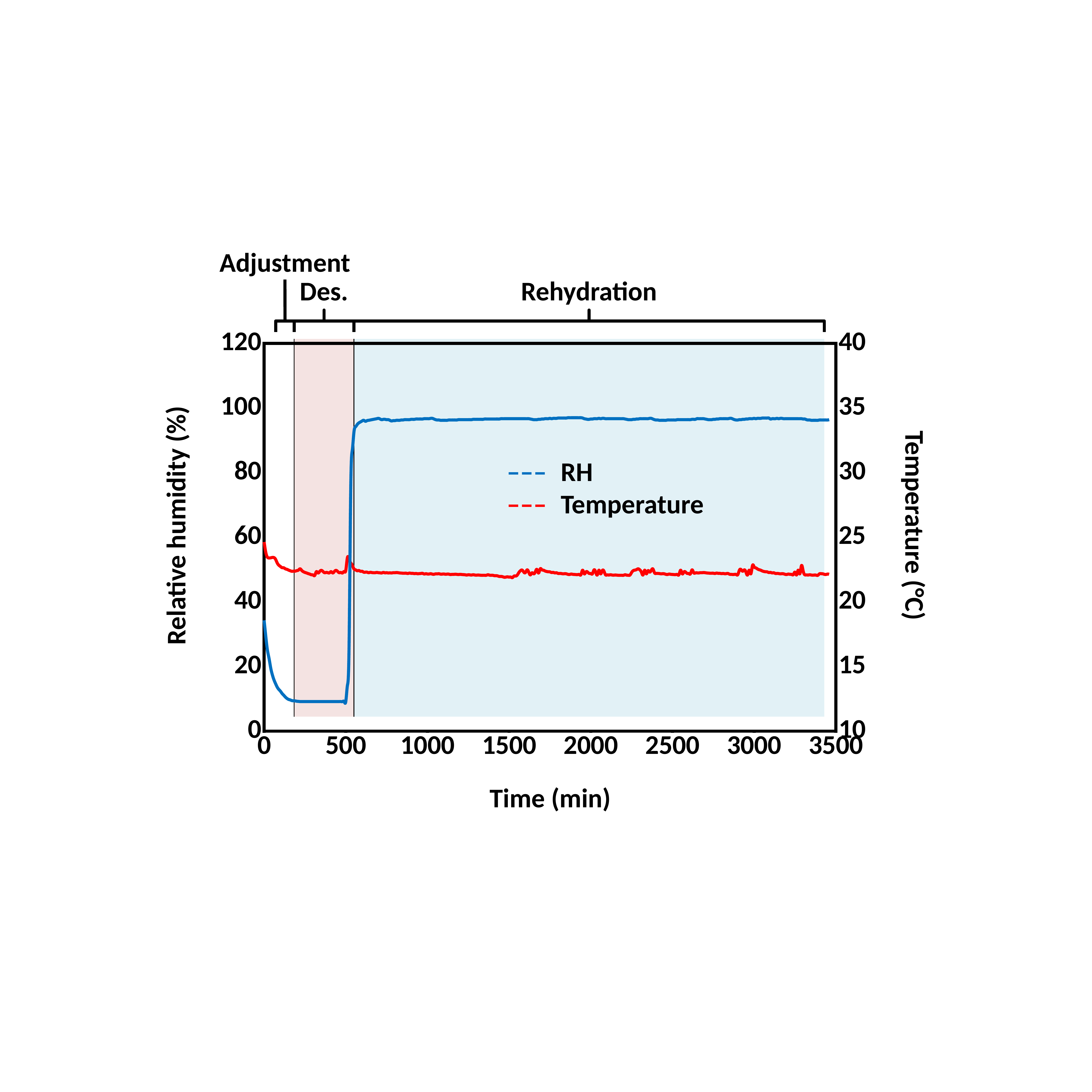

Adjustment
Des.
Rehydration
### Chart
| Category | | |
|---|---|---|
	–––	RH
	 –––	Temperature
Relative humidity (%)
Temperature (°C)
Time (min)
